# Supplementary material for: Proton-transfer-induced 3D/2D hybrid perovskites suppress ion migration and reduce luminance overshoot
Source: Nat Commun. 2020 Jul 6;11:3378. doi: 10.1038/s41467-020-17072-0 (PMC7338442; doi:10.1038/s41467-020-17072-0)
Supplement: Supplementary file 1 — Supplementary Information [file 41467_2020_17072_MOESM1_ESM.pdf]

Supplementary information

Proton-Transfer-Induced 3D/2D Hybrid Perovskites Suppress Ion Migration and Reduce Luminance Overshoot

Hobeom Kim[1,17], Joo Sung Kim[1,17], Jung-Min Heo[1,17], Mingyuan Pei[2], In-Hyeok Park[3], Zhun Liu[4], Hyung Joong Yun[5], Min-Ho Park[1], Su-Hun Jeong[1], Young-Hoon Kim[1], Jin-Woo Park[1], Emad Oveisi[6], Satyawan Nagane[7], Aditya Sadhanala[7,8,16], Lijun Zhang[4], Jin Jung Kweon[9], Sung Keun Lee[9,10], Hoichang Yang[2], Hyun Myung Jang[11], Richard H. Friend[7], Kian Ping Loh[3], Mohammad Khaja Nazeeruddin[12], Nam-Gyu Park[13] and Tae-Woo Lee[1,14,15]*

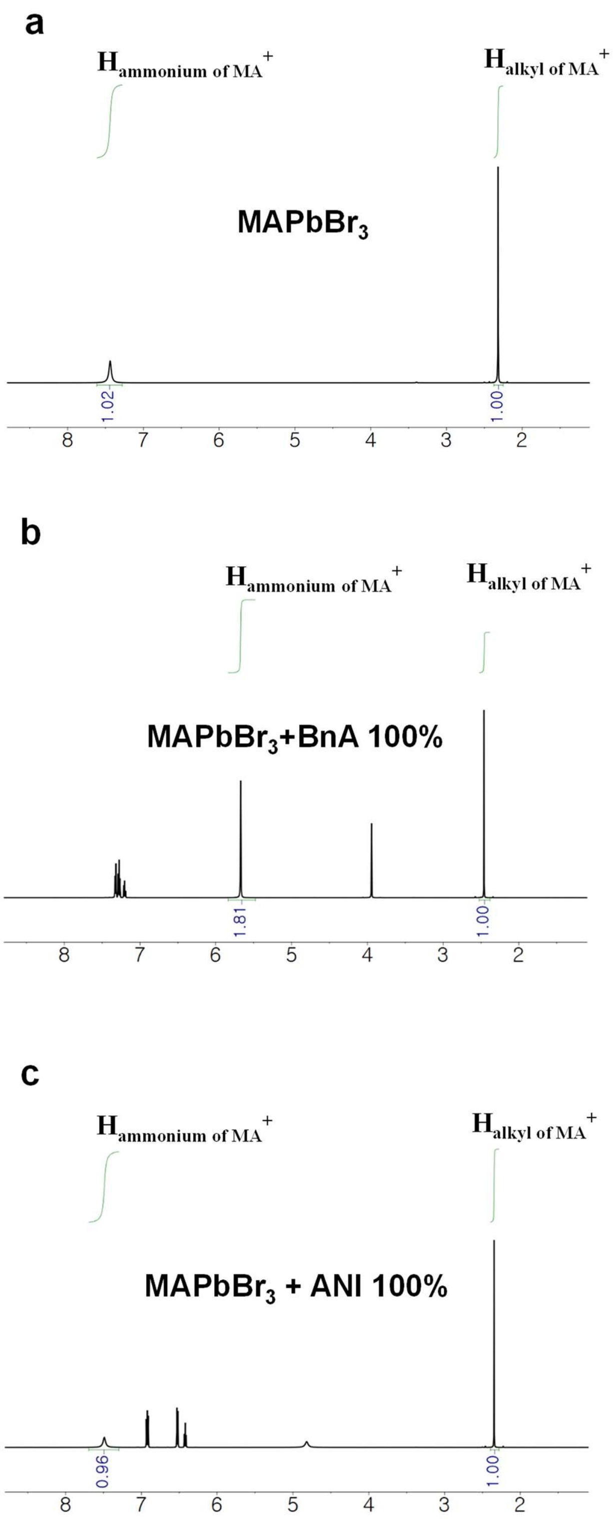

Supplementary Figure 1 ^{1}H NMR spectra of $MAPbBr_3$ solution (a) without additive, (b) with BnA 100 mol%, and (c) ANI 100 mol%.

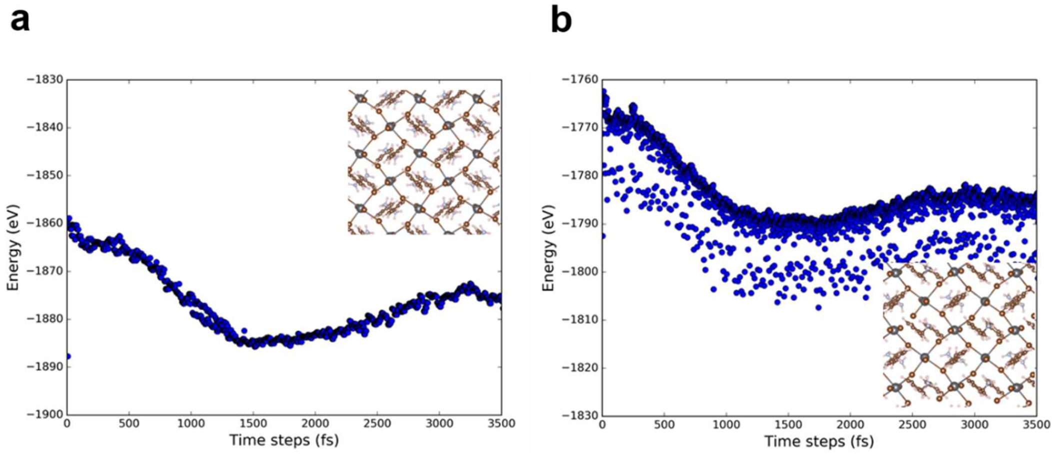

Supplementary Figure 2 Fluctuations of total energy as the evolution of simulation time and the snapshots of atomic configurations after the first-principles molecular dynamics (MD) simulations (3.5 ps) with a time step of 1.2 fs at the temperature of 300 K. **a** and **b** for the 2D perovskites formed by protonated BnA (BnA^+) and unprotonated BnA, respectively. First-principles molecular dynamics (MD) simulations presented the importance of proton-transfer. We utilized a $2\sqrt{2} \times 2\sqrt{2} \times 1$ supercell to compare thermal fluctuation behaviours of perovskite lattice including protonated or unprotonated BnA at 300K. The atoms in the 2D perovskite formed by the protonated BnA (BnA^+) only slightly vibrate around their equilibrium positions and show steady energy fluctuations, which suggests that the 2D structure is well maintained. On the contrary, the 2D perovskite formed by the unprotonated BnA showed highly significant oscillations of the fluctuations of total energy, which indicates the intrinsic instability of the system. This result supports the importance of the protonation which enables BnA^+ to form a strong bond in the lattice and implies that the incorporation of the protonated BnA (BnA^+) can lead to effective passivation since it can maintain the 3D/2D hybrid structure without forming defects in the lattice.

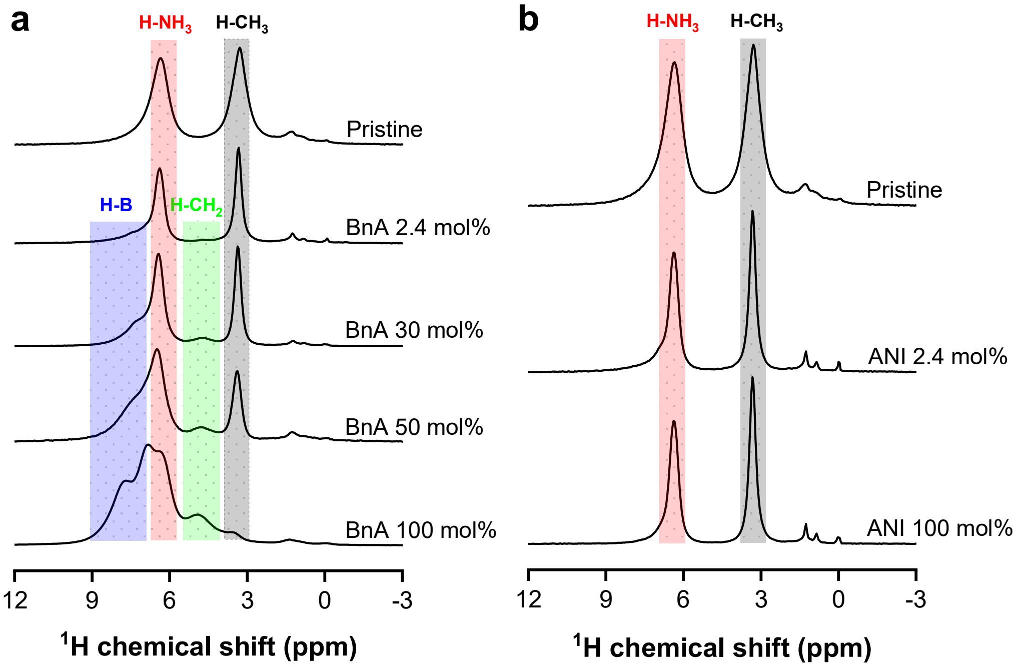

Supplementary Figure 3 The ^{1}H MAS NMR spectra for the solid-state perovskites with varying **a** BnA concentration (0, 2.4, 30, 50 and 100%) and **b** ANI concentration (0, 2.4, and 100%) under a fast spinning speed of 40 kHz at 14.1 T. The pristine sample showed two dominant ^{1}H peaks at 6.36 ppm and 3.30 ppm which can be assigned to the hydrogens in NH_3 (H-NH_3) and CH_3 (H-CH_3) of MA^+, respectively.[1] With the addition of BnA, the spectrum had two more peaks that arose from the hydrogens bonded to the benzene ring (H-B) and in CH_2 (H-CH_2) of BnA^+. The positions of H-B varied in the range of approximately 6.7 to 8 ppm and the peak of H-CH_2 was at around 4.8 ppm.[2] The ^{1}H peak intensity of H-CH_3 decreased with an increase in BnA concentration because MA^+ is deprotonated to be methylamine which can be easily evaporated during the film annealing process. Meanwhile, the peak intensities of H-B, H-NH_3, and H-CH_2 gradually increased confirming that the protonated BnA composes the solid-state crystalline perovskite. Upon the addition of ANI, in contrast, the peak positions of H-NH_3 and H-CH_3 were invariant indicating ANI did not participate in the formation of perovskite.

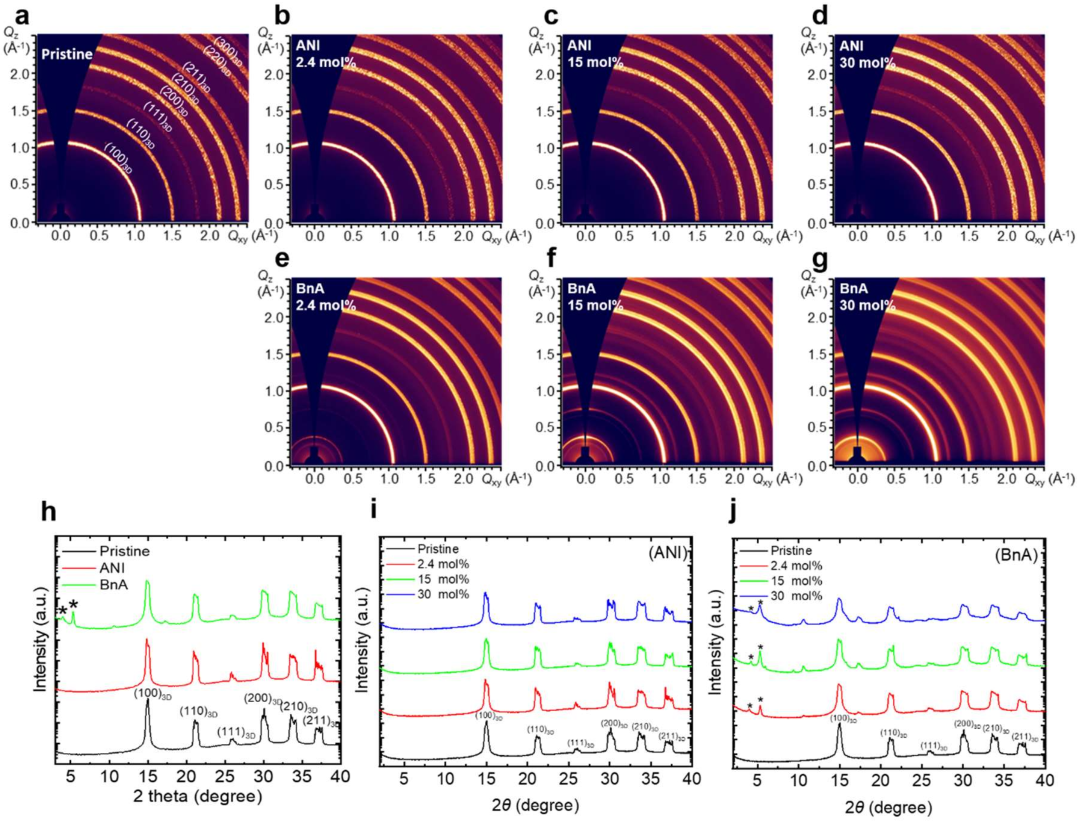

Supplementary Figure 4 (a-g) 2D GIXD patterns of pristine $MAPbBr_3$ film (a), with ANI 2.4 mol% (b), ANI 15 mol% (c), ANI 30 mol% (d), BnA 2.4 mol% (e), BnA 15 mol% (f), and BnA 30 mol% (g). 1D XRD profiles of pristine $MAPbBr_3$ film, $MAPbBr_3$ with ANI 2.4 mol% and BnA 2.4 mol% (h). 1D XRD profiles of $MAPbBr_3$ with different amounts of ANI (i) and BnA (j). Traces have been offset vertically for clarity.

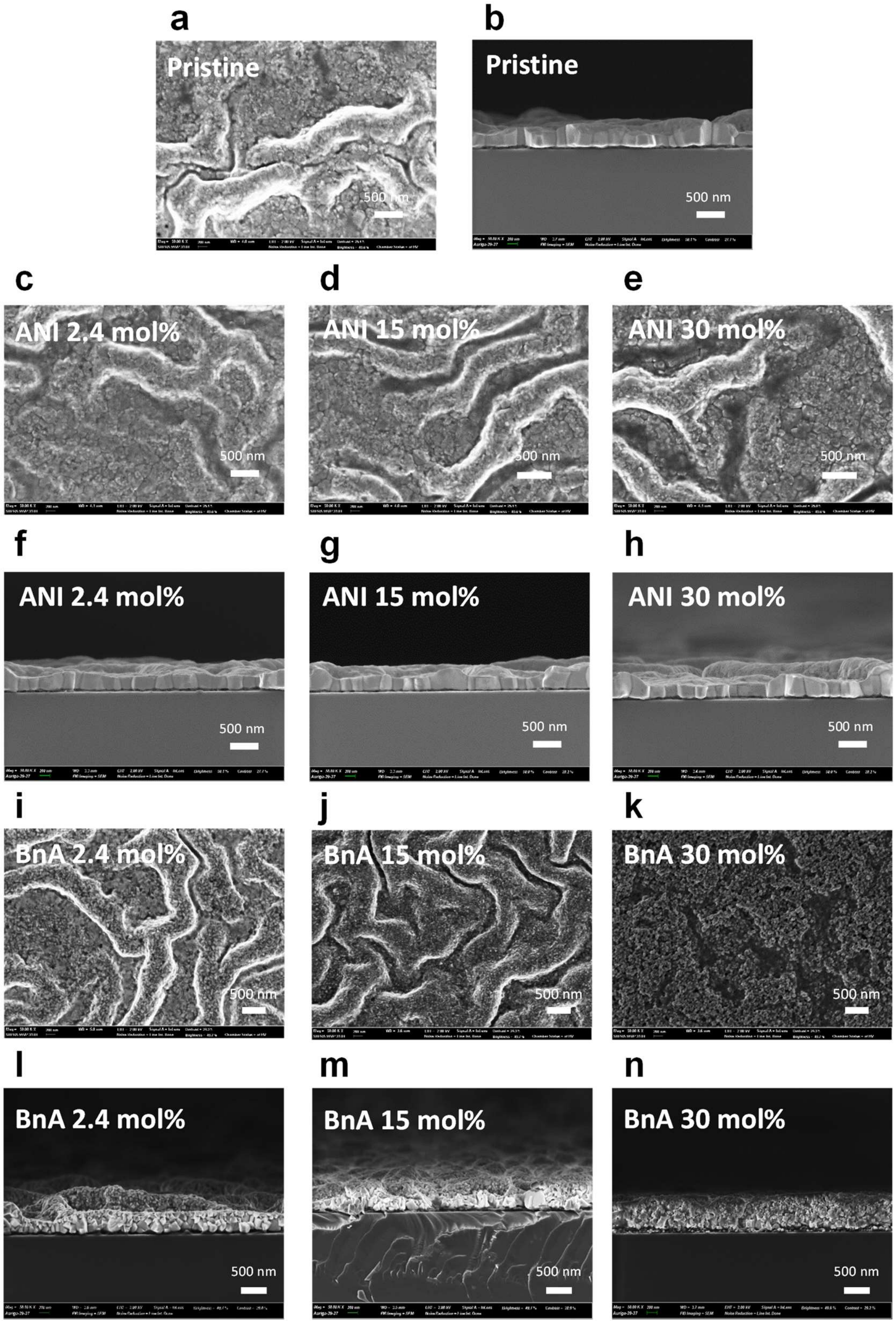

Supplementary Figure 5 Scanning electron microscopic images (SEM) of $MAPbBr_3$ perovskite films. Top-view (a) and cross-sectional view (b) of pristine $MAPbBr_3$, top-view (c-e) and cross-sectional view (f-h) with different amount of ANI, top-view (i-k) and cross-sectional view (l-n) with different amounts of BnA.

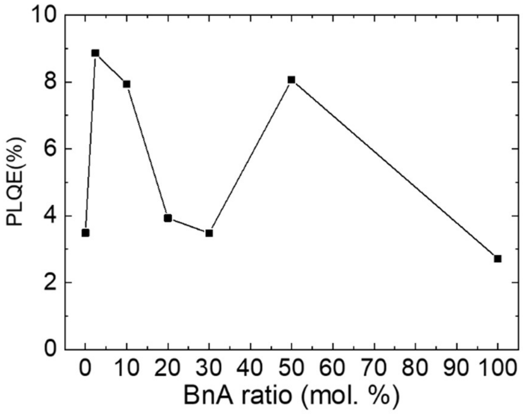

Supplementary Figure 6 PLQE of $MAPbBr_3$ film according to the amount of BnA addition. The films were excited with a continuous-wave 405 nm laser diode (2.5 mW cm^{-2}).

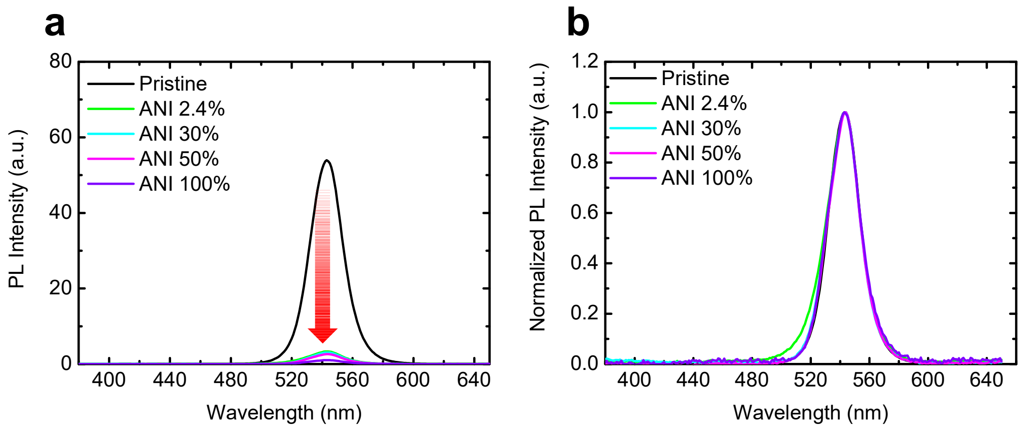

Supplementary Figure 7 Steady-state PL spectra (a) and normalized PL spectra (b) of $MAPbBr_3$ film with different amounts of ANI.

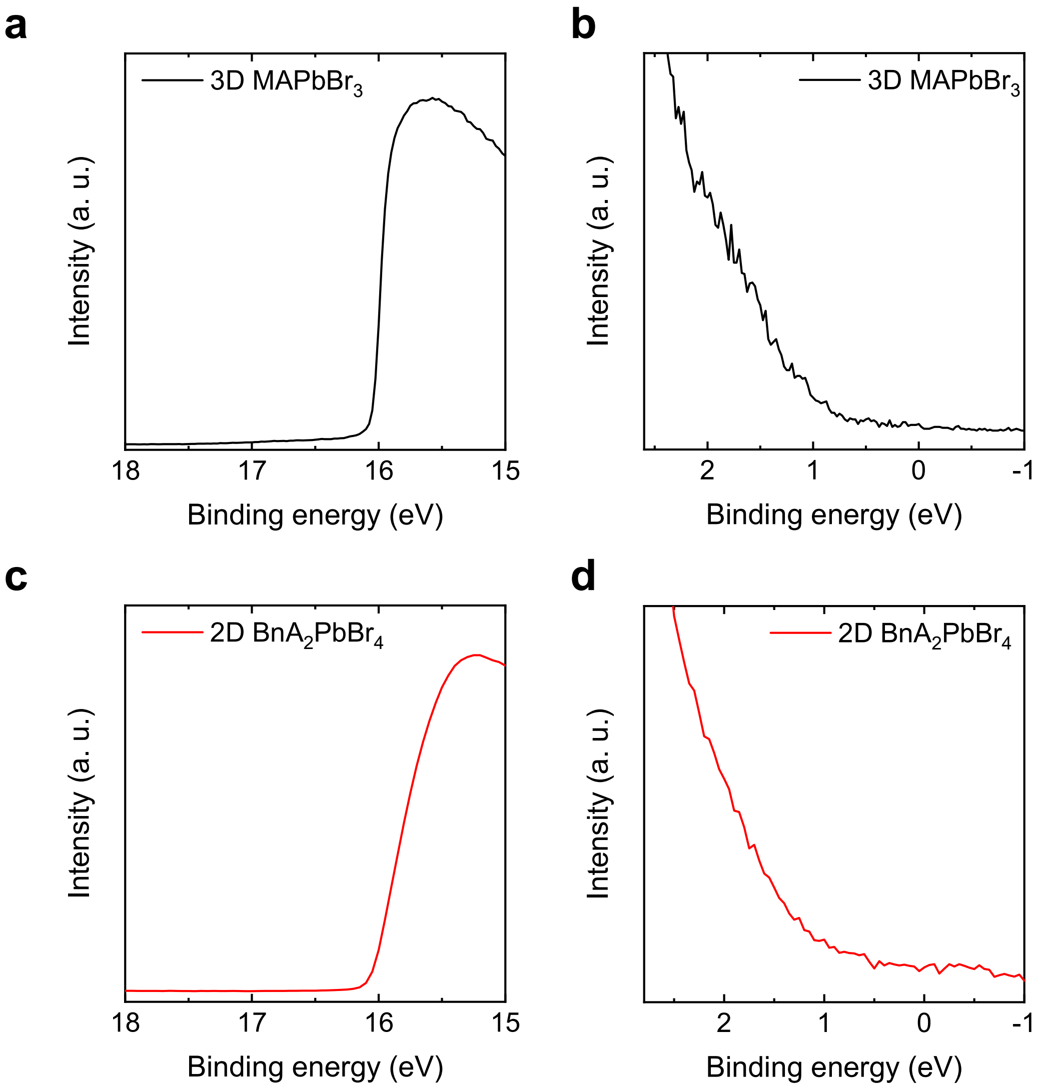

Supplementary Figure 8 UPS spectra of (a, b) 3D $MAPbBr_3$ and (c, d) 2D BnA_2PbBr_4 films showing secondary cut-off (left column) and onset (right column).

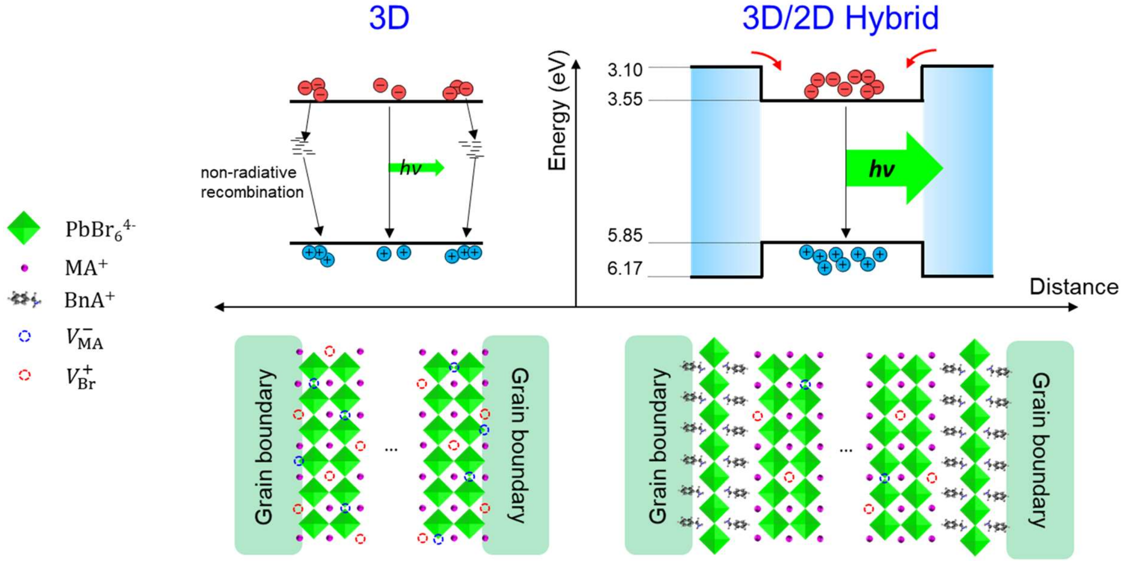

Supplementary Figure 9 Energy band structure of 3D and 3D/2D hybrid perovskites based on UPS result showing recombination behaviour (top) based on the configurations of grain and GB of each perovskite.

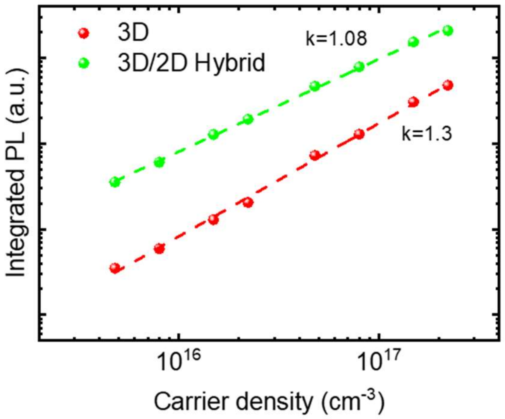

Supplementary Figure 10 Integrated PL as a function of the excitation density of 3D and 3D/2D hybrid perovskites.

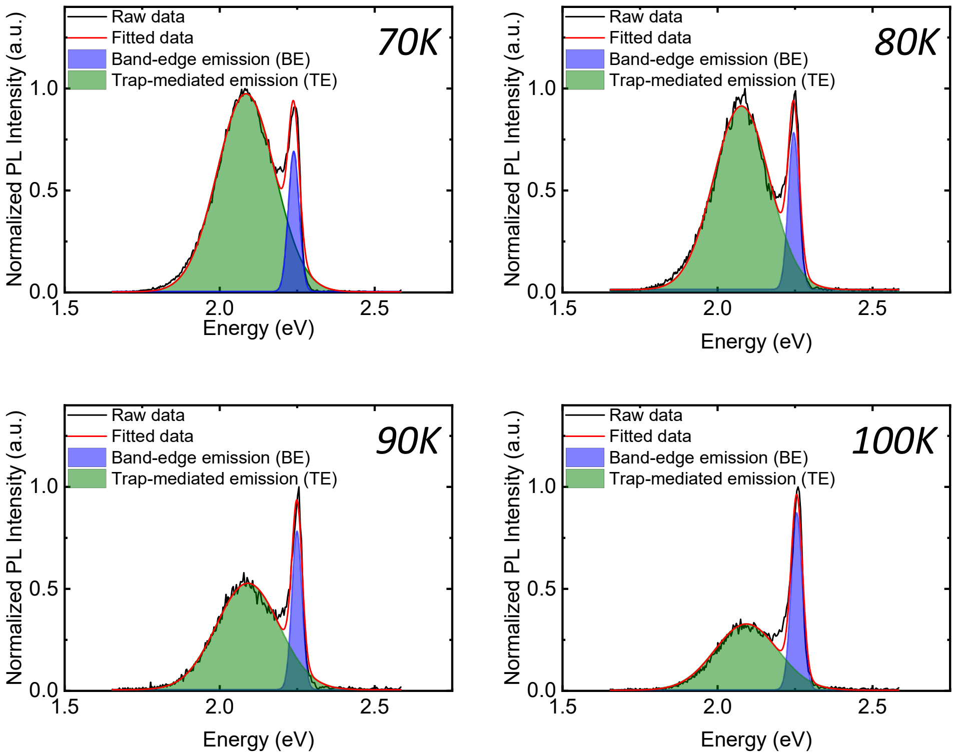

Supplementary Figure 11 PL emission of 3D perovskite, $MAPbBr_3$ according to temperature from 70 K to 100 K. The peaks were deconvoluted into band-edge emission and trap-mediated emission.

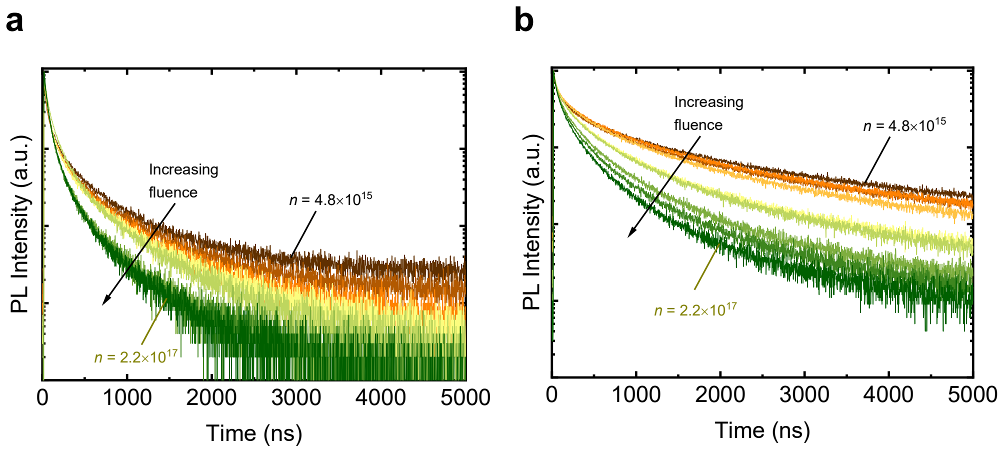

Supplementary Figure 12 TCSPC curves of (a) 3D perovskite and (b) 3D/2D hybrid perovskite as a function of excited carrier density.

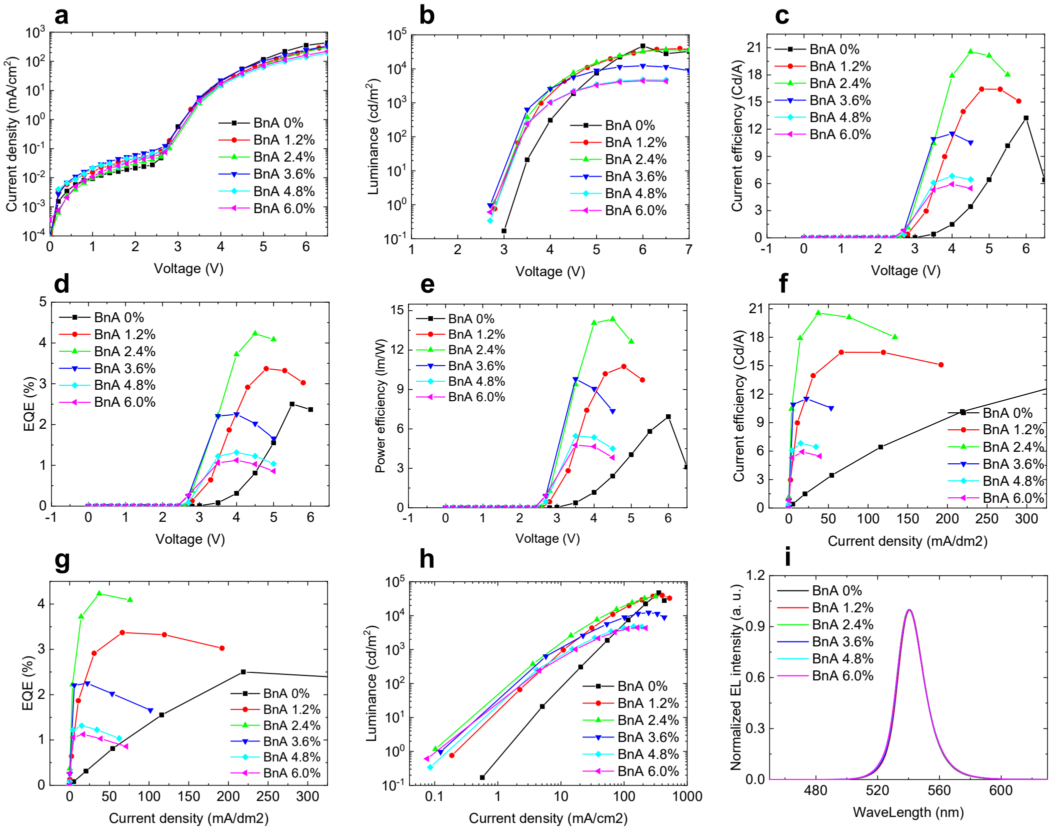

Supplementary Figure 13 Current-voltage-luminance characteristics of PeLEDs with various mol % of BnA. (a) Current density, (b) luminance, (c) current efficiency, (d) external quantum efficiency (EQE), (e) power efficiency of PeLEDs as a function of voltage. (f) Current efficiency, (g) EQE, (h) luminance of PeLEDs as a function of current density. (i) Normalized PL spectra of PeLEDs with various mol% of BnA.

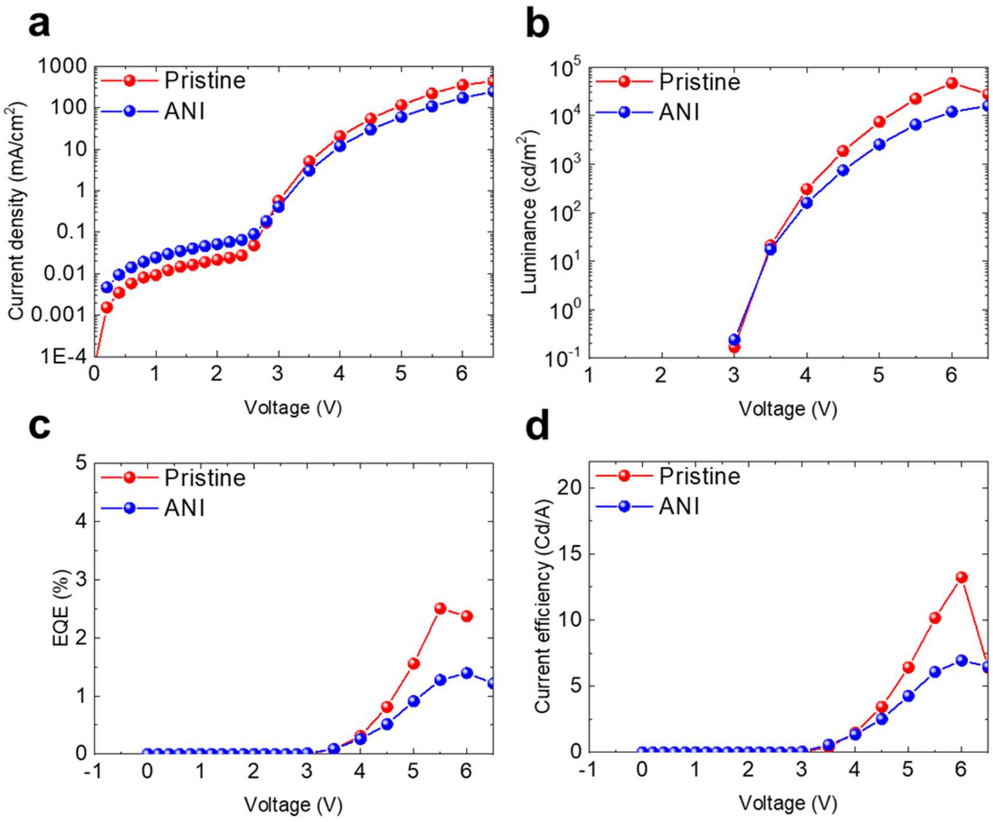

Supplementary Figure 14 EL characteristics of PeLEDs using pristine $MAPbBr_3$ emitter (red) and 2.4 mol % ANI added $MAPbBr_3$ emitter (blue). (a) Current density of PeLEDs as a function of the applied voltage. (b) Luminance of PeLEDs as a function of the applied voltage. (c) External quantum efficiency (EQE) of PeLEDs as a function of the applied voltage. (d) Current efficiency (CE) of PeLEDs as a function of the applied voltage.

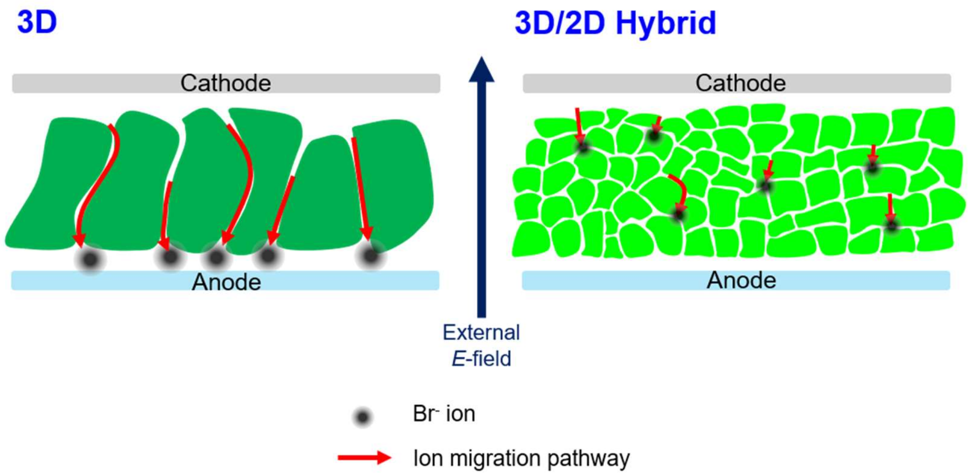

Supplementary Figure 15 Schematic showing ion migration pathway in 3D (left) and 3D/2D hybrid (right) perovskites.

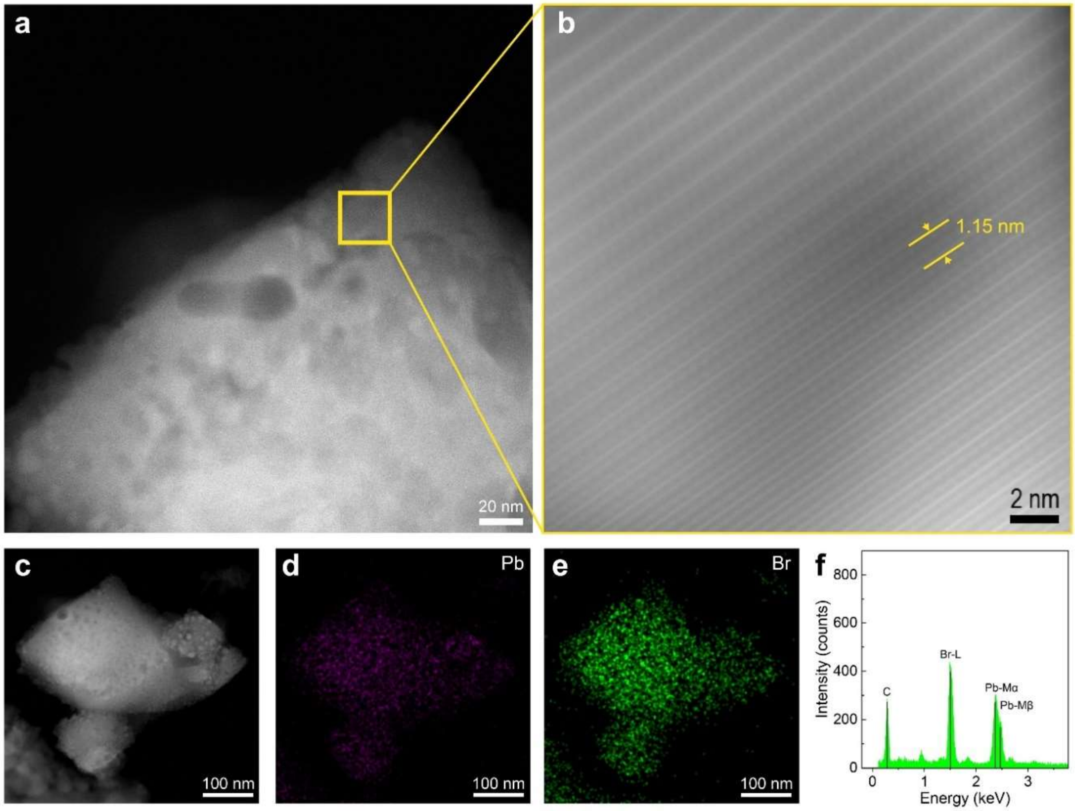

Supplementary Figure 16 (a) HAADF scanning TEM image of a 3D/2D hybrid perovskite grain. (b) High-resolution image of the grain framed with the yellow box in (a) showing the 2D perovskite (n = 1) with d-spacing of 1.15 nm. It is worth noting that the smaller d-spacing (1.15 nm) than that obtained by grazing-incident X-ray diffraction (GIXD) analysis (1.66 nm) can be attributed to transient lattice contraction due to the highly strong beam intensity.[3,4] Interestingly, the highly periodic 2D perovskite was only observable at the shell region of the grain. Because the 2D perovskite has lower surface energy than the 3D perovskite due to its fewer surface dangling bonds and surface relaxation, growth of the 2D perovskite most possibly occurs on the 3D perovskite grains and it also makes an interface with grain boundary which has the highest surface energy, thereby lowering the total potential energy of the system.[3,5] Therefore, the mounted 2D perovskite on the 3D perovskite can effectively passivate the traps and block ion migration. (c) A lower magnification HAADF scanning TEM image of the 3D/2D hybrid perovskite grain shown in (a), corresponding EDXS elemental maps of (d) Pb and (e) Br, and (f) integrated EDX spectrum of the acquired dataset. HAADF-STEM images and corresponding EDXS elemental maps indicate that the grain is composed of Pb and Br elements.

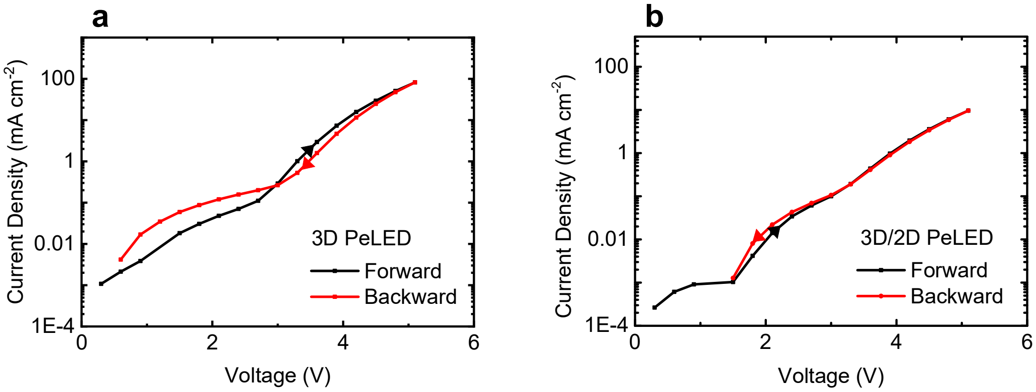

Supplementary Figure 17 Current-voltage hysteresis on forward-backward sweep of (a) 3D PeLED and (b) 3D/2D hybrid PeLED.

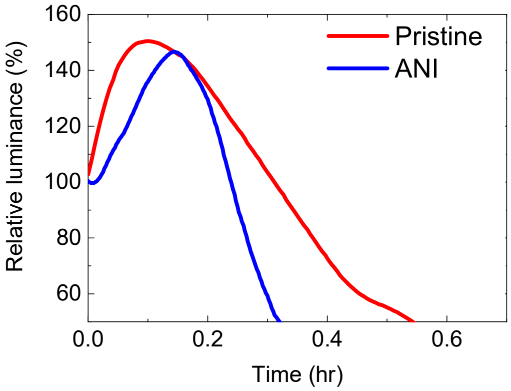

Supplementary Figure 18 Relative luminance of PeLEDs using pristine $MAPbBr_3$ emitter and 2.4 mol % ANI-added $MAPbBr_3$ emitter over time.

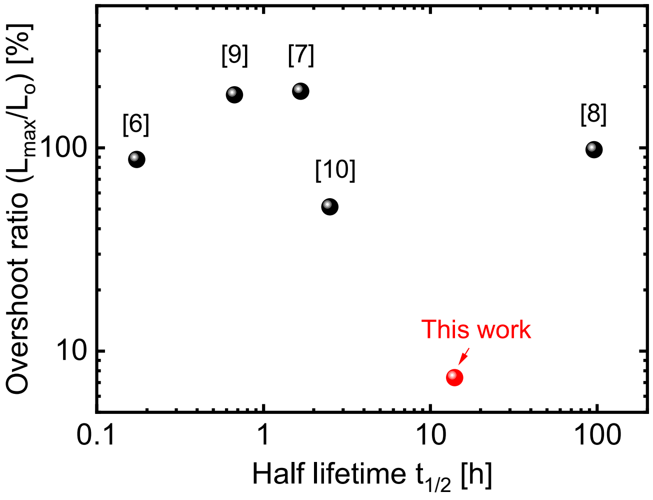

Supplementary Figure 19 A summary of reported luminance overshoot ratio versus half lifetime of PeLEDs with green emission on supplementary table 7. (L_{max}: maximum luminance, L_0: initial luminance)

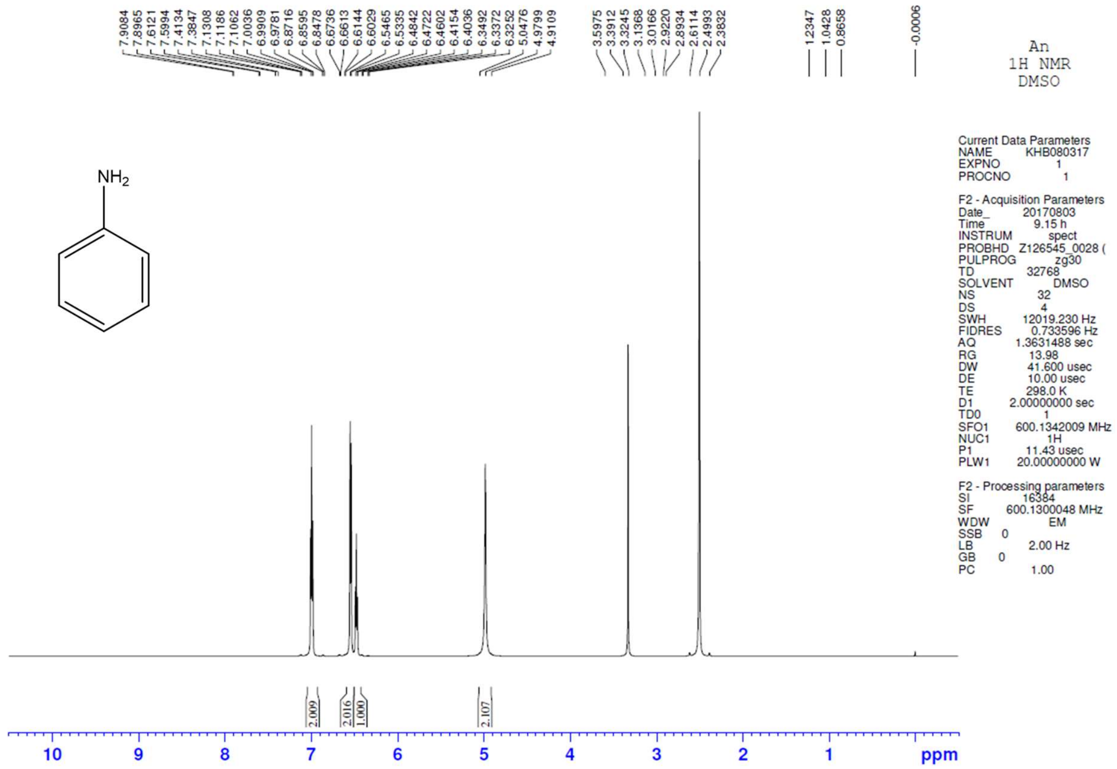

Supplementary Figure 20 ^{1}H-NMR spectrum of ANI in DMSO-d_6.

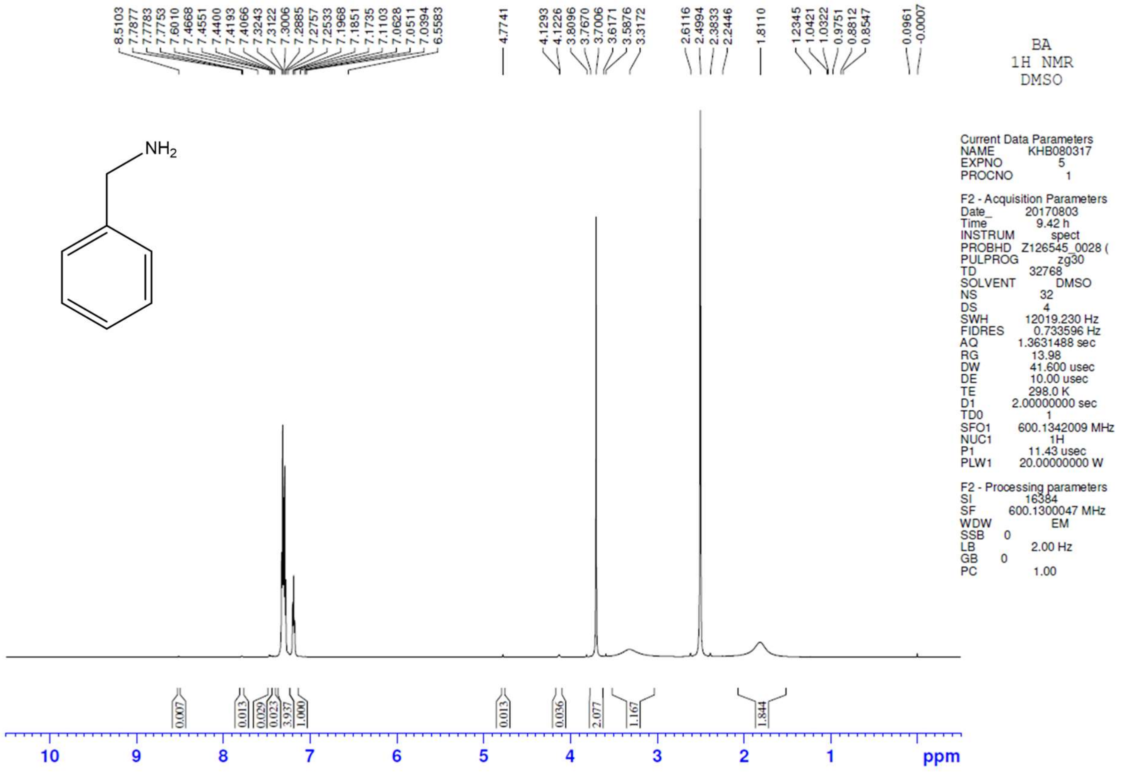

Supplementary Figure 21 ^{1}H-NMR spectrum of BnA in DMSO-d_6.

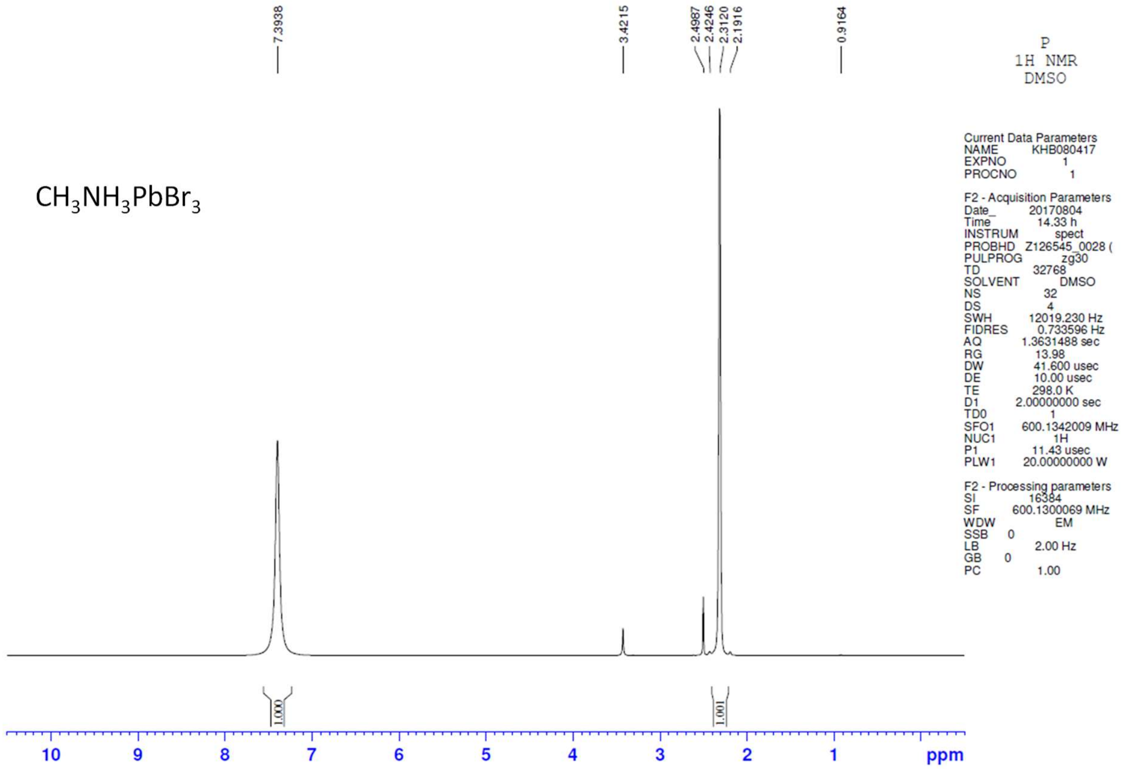

Supplementary Figure 22 ^{1}H-NMR spectrum of $MAPbBr_3$ in DMSO-d_6.

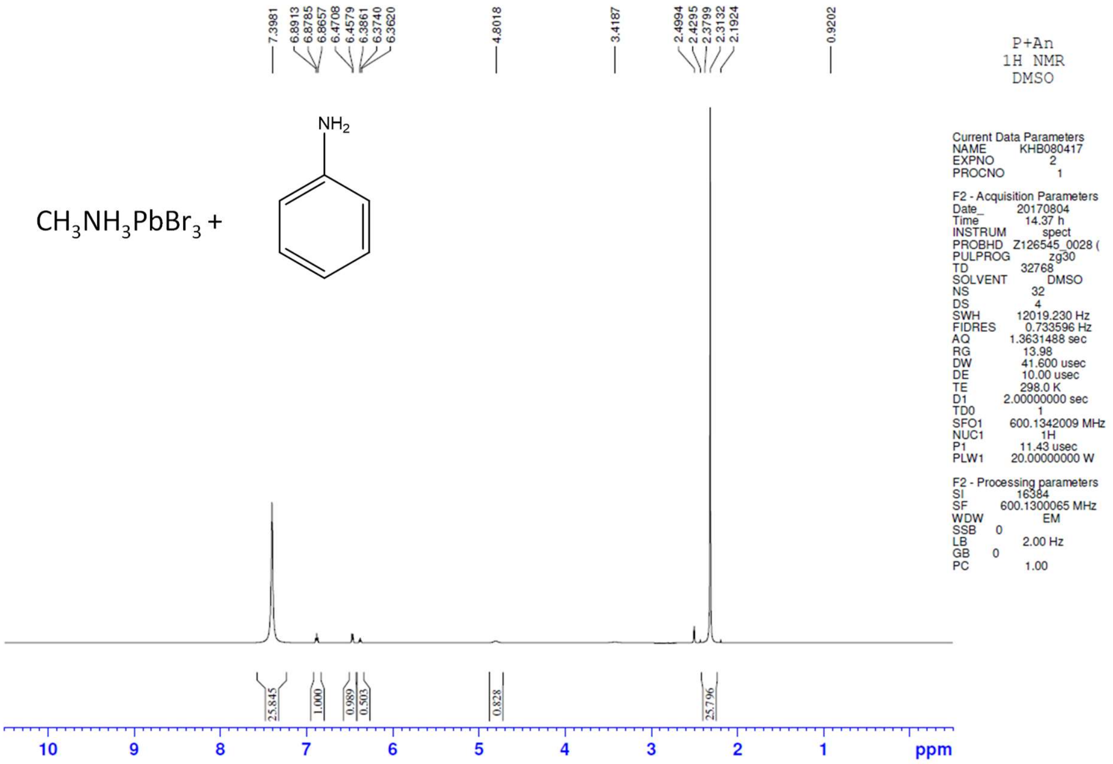

Supplementary Figure 23 ^{1}H-NMR spectrum of $MAPbBr_3$ + 2.4 mol% ANI in DMSO-d_6.

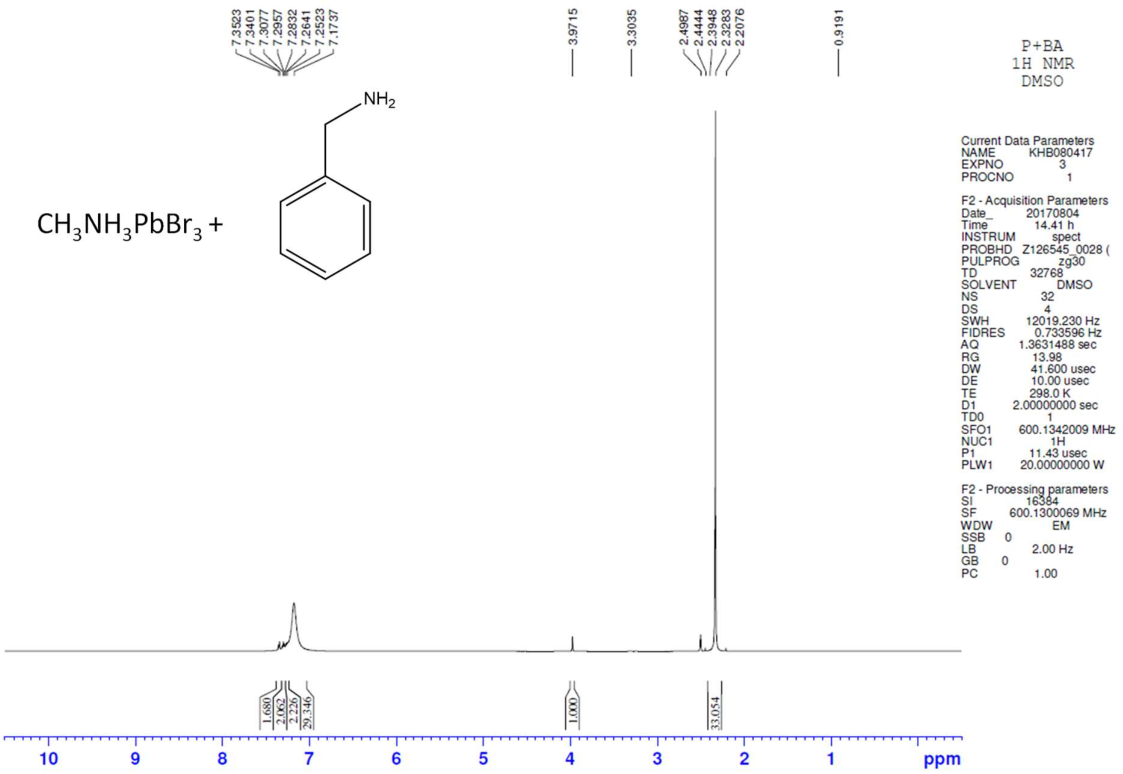

Supplementary Figure 24 ^{1}H-NMR spectrum of $MAPbBr_3$ + 2.4 mol% BnA in DMSO-d_6.

Supplementary Table 1 A summary of integration ratio (*I*) of proton signals ($H_{ammonium}$) / (H_{alkyl}) calculated from ^{1}H NMR spectra of $MAPbBr_3$ solution with additives.

	Integration ratio ($H_{ammonium\ of\ MA}$ /$H_{alkyl\ of\ MA}$)
$MAPbBr_3$	1.02
$MAPbBr_3$ + BnA 100 mol%	1.81
$MAPbBr_3$ + ANI 100 mol%	0.96

Supplementary Table 2 Analysis of the X-ray diffraction spectra in Supplementary Figure 4. The lattice constants of the perovskite crystal were calculated using the Bragg diffraction equation: $2d\sin\theta = n\lambda$ (n = 1, 2, 3···), where d is the crystal plane distance, θ is the diffraction angle at the XRD spectra, n is the order, and λ = 1.54 Å. The lattice constant of the 3D ($n = \infty$) perovskite phase is calculated by (100) peak, resulting in d = 5.89 Å of a cubic $MAPbBr_3$ lattice. The d values of 2D perovskite (n = 1 and n = 2) phases were calculated by additional peaks that emerged at 5.32° and 3.97°, which marked with asterisks in Figure 4h.

Peak	2θ (degree)	d (Å)
$(100)_{3D}$	15.02	5.89
$(110)_{3D}$	21.13	4.20
$(111)_{3D}$	26.02	3.42
$(200)_{3D}$	30.16	2.96
$(210)_{3D}$	33.52	2.67
$(211)_{3D}$	37.27	2.41
$(220)_{3D}$	43.24	2.09
$(300)_{3D}$	45.29	2.00
$(002)_{n=1}$	5.32	16.58
$(002)_{n=2}$	3.97	22.21

Supplementary Table 3 Bi-exponential decay function, $y = A_1e^{-t/\tau_1}+A_2e^{-t/\tau_2}$ fitted results of PL lifetime curves of pristine, 2.4 mol% ANI-added, and 2.4 mol% BnA-added $MAPbBr_3$ films on Quartz/Buf-HIL substrates.

Condition	A_1	τ_1 [ns]	A_2	τ_2 [ns]	τ_{Ave} [ns]	χ^2
Pristine	326.3	119.2	540.6	25.8	60.94	0.967
ANI	438	37.8	463.0	12.3	24.71	0.997
BnA	354.0	487.6	480.8	82.7	254.4	1.048

Supplementary Table 4 Summarized result of UPS analysis of $MAPbBr_3$ and BnA_2PbBr_4.

	Secondary cut-off [eV]	Onset [eV]	IP [eV]
$MAPbBr_3$	16.08	0.71	5.85
BnA_2PbBr_4	16.04	0.99	3.10

Supplementary Table 5 Summarized electrical and luminance characteristics of PeLEDs based on $MAPbBr_3$ with various mol% of BnA.

	BnA (mol%)					
Parameter	0	1.2	2.4	3.6	4.8	6.0
Turn-on Voltage [V] @ 1cd m^{-2}	3.18	2.83	2.77	2.70	2.83	2.76
Max. CE [cd A^{-1}]	13.23 @ 6 V	16.42 @ 4.8 V	20.55 @ 4.5 V	11.52 @ 4 V	6.82 @ 4 V	5.93 @ 4 V
Max. PE [lm W^{-1}]	6.92 @ 6 V	10.75 @ 4.8 V	14.35 @ 4.5 V	9.80 @ 3.5 V	5.46 @ 3.5 V	4.76 @ 3.5 V
Max. L [cd m^{-2}]	46920	39469	36656	12294	4777	4431

Supplementary Table 6 Summarized energy barrier of ion migration for each pathway in 3D and 3D/2D hybrid perovskites.

**Pathways of ion migration in 3D perovskite**	**Energy barrier for ion migration**	**Pathways of ion migration in 3D/2D hybrid perovskite**
Intra grain **(3D → 3D)**	≈	Intra grain **(3D → 3D)**
Grain bulk to grain surface **(3D → 3D)**	<	Grain bulk to grain surface **(3D → 2D)**
On grain boundary (grain surface)	<	On grain boundary (grain surface)

Supplementary Table 7 A summary of published luminance overshoot of PeLEDs with visible emission. (T_{50}: half lifetime, L_0: initial luminance, [a] PMMA: poly(methyl methacrylate), [b] B3PYMPM: 4,6-bis(3,5-di(pyridin-3-yl)phenyl)-2-methylpyrimidine, [c] Bphen: Bathophenanthroline, [d] SOCP: Self-organized conducting polymer)

Ref.	Device structure	Emission layer (colour)	Operational lifetime	Overshoot ratio (L_{max}/L_0) [%]
6	ITO/PEDOT:PSS/Perovskite/ PMMA[a]/B3PYMPM[b]/LiF/Al	$CsPbBr_3/MABr$ (Green)	T_{50}≈10.42 m	87.6 at 166.67 mA cm^{-2}
7	ITO/NiO_x/Perovskite/TPBi/LiF/Al	$FAPbBr_3/BnABr$ (Green)	T_{50}≈1.67 h	189.8 L_0≈100 cd m^{-2}
8	ITO/LiF/Perovskite /LiF/Bphen[c]/LiF/Al	$CsPbBr_3$ (Green)	T_{50}≈96 h	97.9
9	SOCP[d]/Perovskite/TPBi/LiF/Al	$FA_xCs_{1-x}PbBr_3$ (Green)	T_{50}≈0.67 h	182.2 L_0≈100 cd m^{-2}
10	SOCP/Perovskite/TPBi/LiF/Al	$MAPbBr_3$:TPBI (Green)	T_{50}≈2.5 h	51.2 L_0≈100 cd m^{-2}
**This work**	**FTO/Buf-HIL/Perovskite /TPBi/LiF/Al**	**$MAPbBr_3$: $BnA_2MA_{n-1}Pb_nBr_{3n+1}$ (n=1 or 2) (Green)**	**T_{50}≈14 h**	**7.4 L_0≈100 cd m^{-2}**

Supplementary Note 1: Remarkably enhanced protonation tendency of benzylamine (BnA)

The equilibrium constant of proton transfer between methylamine (MA) and benzylamine (BnA) can be written as

$$K_{\mathrm{BnA}} = \frac{[\mathrm{MA}][\mathrm{BnAH}^+]}{[\mathrm{MAH}^+][\mathrm{BnA}]} = \frac{10^{-4.66}}{10^{-3.38}} = 10^{-1.28}, \qquad \text{(Supplementary Equations 1)}$$

where $'10^{-3.38}'$ denotes the equilibrium constant of protonation between MA and MAH^+.[11] The equilibrium constant of the proton transfer between MA and aniline (ANI) is given by

$$K_{\mathrm{ANI}} = \frac{[\mathrm{MA}][\mathrm{ANIH}^+]}{[\mathrm{MAH}^+][\mathrm{ANI}]} = \frac{10^{-9.40}}{10^{-3.38}} = 10^{-6.02}, \qquad \text{(Supplementary Equations 2)}$$

so the ratio of these two equilibrium constants is

$$K_{\mathrm{R}} = \frac{K_{\mathrm{BnA}}}{K_{\mathrm{ANI}}} = \frac{10^{-1.28}}{10^{-6.02}} = 5.5 \times 10^4. \qquad \text{(Supplementary Equations 3)}$$

Thus, the protonated BnA (i.e., $BnAH^+$) predominates over the protonated ANI with the standard Gibbs free-energy change ΔG^o = -6.51 kcal·mol^{-1} at 300 K. This result thermo-dynamically supports our conclusion in the main text that the protonation tendency of BnA is much (> 10^4 times) stronger than that of ANI.

Supplementary Note 2: Improved PL of the 3D/2D hybrid perovskites owing to the energy band alignment

Alignment of energy bands contributes to the improvement in PL properties of the 3D/2D hybrid perovskite film. Ultraviolet photoemission spectroscopy (UPS) analysis of each 3D $MAPbBr_3$ and 2D BnA_2PbBr_4 film confirmed the type-I quantum well band alignment of 3D/2D hybrid perovskite (Supplementary Figure 8 and Supplementary Table 4).[12,13] In this quantum well, the excited charge carriers can be efficiently confined within the 3D phase (Supplementary Figure 9), enabling efficient radiative recombination in 3D/2D hybrid perovskite without a shift of emission wavelength.[14] In addition, the 2D perovskite reduces the defect density by passivating dangling bonds on 3D perovskite grains, which can act as charge trap sites leading to the increased rate of non-radiative recombination.

Supplementary References

1. Anelli, C. *et al.* Investigation of Dimethylammonium Solubility in $MAPbBr_3$ Hybrid Perovskite: Synthesis, Crystal Structure, and Optical Properties. *Inorg. Chem.* **58**, 944–949 (2019).
2. Fergoug, T. & Bouhadda, Y. Determination of Hassi Messaoud asphaltene aromatic structure from ^1H & ^{13}C NMR analysis. *Fuel* **115**, 521–526 (2014).
3. Ferguson, K. R. *et al.* Transient lattice contraction in the solid-to-plasma transition. *Sci. Adv.* **2**, e1500837 (2016).
4. Maehlen, J. P., Mongstad, T. T., You, C. C. & Karazhanov, S. Lattice contraction in photochromic yttrium hydride. *J. Alloys Compd.* **580**, S119–S121 (2013).
5. Yang, Y., Gao, F., Gao, S. & Wei, S.-H. Origin of the stability of two-dimensional perovskites: a first-principles study. *J. Mater. Chem. A* **6**, 14949–14955 (2018).
6. Lin, K. *et al.* Perovskite light-emitting diodes with external quantum efficiency exceeding 20 per cent. *Nature* **562**, 245–248 (2018).
7. Lee, S. *et al.* Control of Interface Defects for Efficient and Stable Quasi-2D Perovskite Light-Emitting Diodes Using Nickel Oxide Hole Injection Layer. *Adv. Sci.* **5**, 1801350 (2018).
8. Shi, Y. *et al.* A Strategy for Architecture Design of Crystalline Perovskite Light-Emitting Diodes with High Performance. *Adv. Mater.* **30**, 1800251 (2018).
9. Cho, H. *et al.* High-Efficiency Polycrystalline Perovskite Light-Emitting Diodes Based on Mixed Cations. *ACS Nano* **12**, 2883–2892 (2018).
10. Park, M. *et al.* Efficient Perovskite Light-Emitting Diodes Using Polycrystalline Core–Shell-Mimicked Nanograins. *Adv. Funct. Mater.* 1902017 (2019).
11. Gary O. Spessard. Introduction to organic chemistry. *J Chem Educ* **54**, A294 (1977).

12. Zou, W. *et al.* Minimising efficiency roll-off in high-brightness perovskite light-emitting diodes. *Nat. Commun.* **9**, 608 (2018).

13. Yang, X. *et al.* Efficient green light-emitting diodes based on quasi-two-dimensional composition and phase engineered perovskite with surface passivation. *Nat. Commun.* **9**, 570 (2018).

14. Wang, Z. *et al.* Efficient ambient-air-stable solar cells with 2D–3D heterostructured butylammonium-caesium-formamidinium lead halide perovskites. *Nat. Energy* **2**, 17135 (2017).
